# Spectroscopic mapping and selective electronic tuning of molecular orbitals in phosphorescent organometallic complexes – a new strategy for OLED materials

**DOI:** 10.3762/bjnano.5.234

**Published:** 2014-11-26

**Authors:** Pascal R Ewen, Jan Sanning, Tobias Koch, Nikos L Doltsinis, Cristian A Strassert, Daniel Wegner

**Affiliations:** 1 Institut for Molecules and Materials, Radboud Universiteit Nijmegen, P.O. Box 9010, 6500 GL Nijmegen, The Netherlands; 2 Physikalisches Institut, Westfälische Wilhelms Universität Münster, Wilhelm-Klemm-Straße 10, 48149 Münster, Germany; 3 Institut für Festkörpertheorie and Center for Multiscale Theory and Computation, Westfälische Wilhelms Universität Münster, Wilhem-Klemm-Straße 10 and Corrensstraße 40, 48149 Münster, Germany

**Keywords:** charge transfer, density-functional theory, frontier orbitals, hybridization, OLED, Pt(II) complex, scanning tunneling microscopy, scanning tunneling spectroscopy, triplet emitters

## Abstract

The improvement of molecular electronic devices such as organic light-emitting diodes requires fundamental knowledge about the structural and electronic properties of the employed molecules as well as their interactions with neighboring molecules or interfaces. We show that highly resolved scanning tunneling microscopy (STM) and spectroscopy (STS) are powerful tools to correlate the electronic properties of phosphorescent complexes (i.e., triplet emitters) with their molecular structure as well as the local environment around a single molecule. We used spectroscopic mapping to visualize several occupied and unoccupied molecular frontier orbitals of Pt(II) complexes adsorbed on Au(111). The analysis showed that the molecules exhibit a peculiar localized strong hybridization that leads to partial depopulation of a d_z²_ orbital, while the ligand orbitals are almost unchanged. We further found that substitution of functional groups at well-defined positions can alter specific molecular orbitals without influencing the others. The results open a path toward the tailored design of electronic and optical properties of triplet emitters by smart ligand substitution, which may improve the performance of future OLED devices.

## Introduction

Organic light emitting diodes (OLEDs) based on phosphorescent Ir(III) or Pt(II) complexes (also referred to as triplet emitters) are a very promising alternative to current devices for highly efficient lighting and display technologies [1]. In the quest to improve OLEDs, a fundamental understanding of the nature and interactions of the involved molecular orbitals (MO) is crucial both within each organic layer and at the interfaces of the multilayer device [2]. Cyclic voltammetry (CV) is widely used to determine the oxidation and reduction potentials of organometallic molecules and has rightfully become a very popular technique for electrochemical studies [3–4]. However, interactions of the molecules with their environment (e.g., host–guest interactions, hybridization at surfaces and interfaces, interaction in aggregates) can significantly change the energetic position and order of molecular orbitals, but CV cannot always provide information on such effects whenever the local environment is not well known. Moreover, CV depends delicately on many parameters and necessitates great care during execution and analysis [5], but the major popularity of CV and its transformation as a quick tool in many labs entails the risk of disregarding this [6].

Looking at alternative surface science-based methods, photoemission and inverse photoemission spectroscopy techniques are common to address the electronic properties of molecular systems under well defined conditions [7–8]. As a drawback, these methods are each limited to the occupied or unoccupied states, respectively. Moreover, in a structurally complex or inhomogeneous sample the spectra display the average of distributed MO levels due to a lack of spatial resolution. This has led to controversies as to how the MO levels should be deduced from the spectra [9–10]. In this context, the combined power of atomic and high energy resolution in scanning tunneling microscopy (STM) and spectroscopy (STS) makes it an ideal tool to study the electronic properties of adsorbed molecules with precise knowledge and control of the local environment around a single molecule. Although this method is limited to an energy range a few eV around the Fermi energy *E*_F_, this is usually sufficient to probe the relevant frontier orbitals [11–15]. Several studies have performed STM and STS on organometallic compounds, mainly on porphyrins and phthalocyanines [16–22]. Considering this general success, it is surprising that phosphorescent complexes have barely been investigated via scanning probe methods. Almost all studies are limited to the analysis of thin film and crystal growth of Pt(II) or Ir(III) complexes via atomic force microscopy [23–24] or STM [25–28] and lack the submolecular resolution to address specific parts of a molecule. Only a single study employed STS [29], but without showing STM images or stating where on the molecule the data had been acquired. Essentially, prior to our involvement [30] no publication has utilized the advantages of combined STM and STS to study triplet emitters.

**Figure 1:**
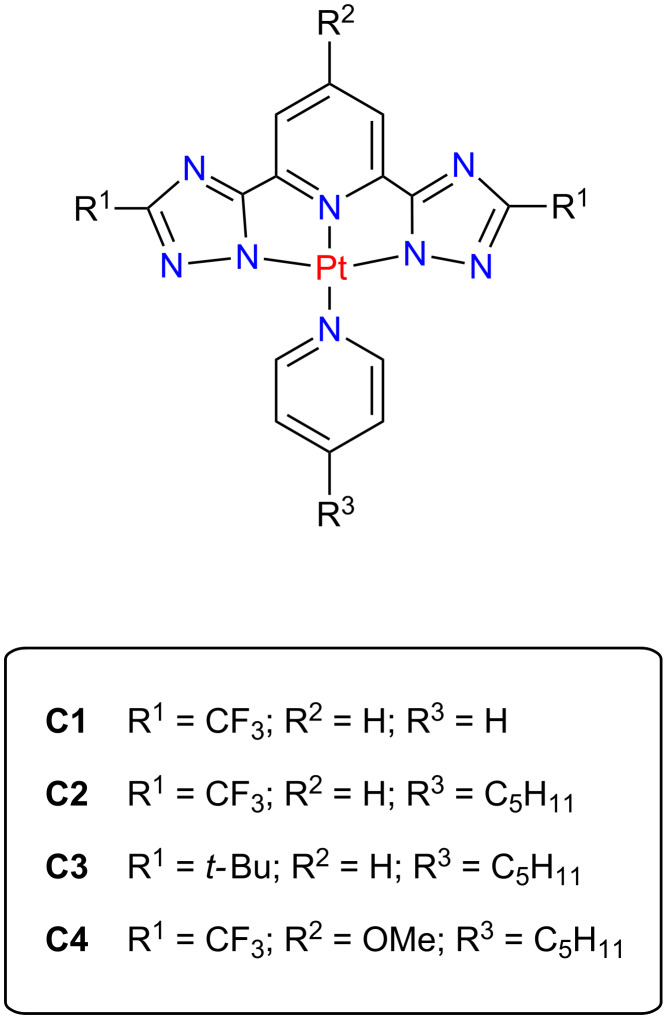
Molecular structure of the complexes **C1** to **C4**. In all cases the Pt atom is fourfold coordinated by N atoms, stemming from a tridendate ligand (TL, containing two triazole groups and one pyridine) and an ancillary ligand (AL, containing a pyridine group). The substituents R^1^ to R^3^ are varied in order to investigate their influence on the adsorption as well as the electronic structure.

We have performed STM and STS measurements at cryogenic temperatures on submonolayer amounts of various square-planar Pt(II) complexes on a Au(111) single-crystal surface. These complexes coordinate a Pt atom to a tridentate ligand (TL, with substituents R^1^ and R^2^) and an ancillary ligand (AL, substituent R^3^), see Figure 1, and are known to be highly efficient (phosphorescent) triplet emitters both in monomeric and aggregated form [31–32]. We identified a number of occupied and unoccupied frontier orbitals. Comparison with density functional theory (DFT) calculations allows the unambiguous assignment of all MOs from the HOMO–2 to the LUMO+2. We found that the complexes show a peculiar site-specific hybridization to the Au(111) substrate that only involves the Pt atom but leaves the ligand orbitals essentially unaltered. We also show that different substituents at particular positions of the molecular structure alter the HOMO and LUMO levels, and we propose a strategy of fine-tuning both levels independently, which should permit the tunability of the HOMO–LUMO gap (and thus the emission color) as well as charge-injection barriers in a device.

## Results and Discussion

### Methods and sample preparation

The experiments were performed under ultrahigh vacuum conditions (base pressure <10^−10^ mbar) using a commercial low-temperature scanning tunneling microscope (Createc LT-STM). The synthesis of the complexes is described elsewhere [32–33]. The sample preparation was done by repeated sputter-annealing cycles of the Au(111) single-crystal substrate followed by thermal evaporation of the molecules from a commercial evaporator (Createc TUBOmini) at about 420 K to 470 K, while the substrate was held at room temperature. Typical deposition times were on the order of 20 to 30 seconds, leading to sub-monolayer coverage on the metal surface. Subsequently, the sample was transferred in situ into the cold STM (*T* = 5 K).

All images where taken in constant-current mode. For the tunneling spectra the current *I* and the differential conductance *dI*/*dV* (via lock-in technique, modulation voltage 10–20 mV) were measured simultaneously as a function of sample bias *V* under open-feedback conditions. The bias voltage is always given with respect to the sample, i.e., positive sample bias corresponds to electrons tunneling from occupied electronic states of the tip into unoccupied states of the sample, and *V* = 0 corresponds to the Fermi energy *E*_F_. In good approximation, *dI*/*dV* is proportional to the local density of states of the sample. Energy-resolved spectral maps (that visualize the spatial distribution of molecular orbitals) were acquired by measuring *dI*/*dV* at a fixed bias as a function of lateral position in constant-current mode.

For the DFT calculations shown here, Kohn–Sham molecular orbitals were calculated in the gas phase with the Gaussian 09 package [34] using the PBE0 hybrid exchange-correlation functional [35] and the SDD basis set [36]. The molecular orbitals were visualized using the VMD 1.9 software. The orbital energies in the gas-phase calculations are computed with respect to the vacuum level. For a comparison with the measured values from STS (which are given relative to *E*_F_), a constant corresponding to the work function of 5.1 eV has to be added to the calculated values (cf. details in the discussion).

### Structural analysis

**Figure 2:**
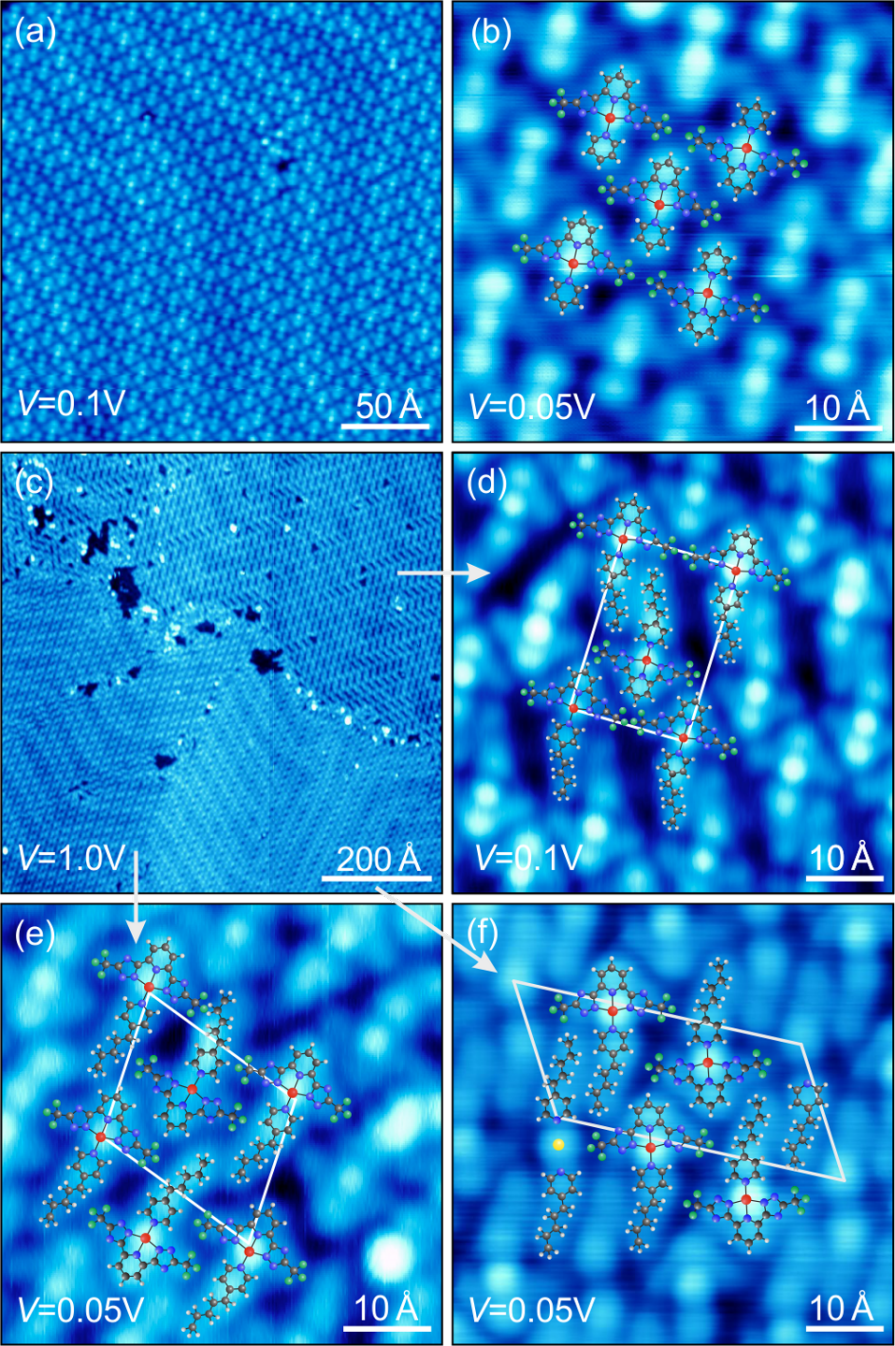
Topography analysis of a monolayer of **C1** (a,b) and **C2** (c–f) on Au(111). **C1** grows in only one close-packed structure probably due to steric packing. **C2** shows three different ordered structures, indicating the additional role of van der Waals forces between neighboring R^3^ alkyl chains for the self-assembly.

For the the structural characterization of as-grown molecular films, we focus on the two Pt complexes **C1** and **C2** (see Figure 1). STM images of the first monolayer of **C1** (Figure 2a,b) and **C2** (Figure 2c–f) on Au(111) reveal that the underlying Au(111) herringbone reconstruction is essentially unaffected by the adsorbed layer. This is indicative of an overall weak adsorbate-substrate interaction [37]. The close-up images exhibit submolecular resolution and clearly reflect the chemical building blocks. By superimposing the corresponding molecular structures we can attribute the highest round protrusions to the Pt atom in the center of the complexes. This bright feature is surrounded by the TL that appears as a slightly dimmer protrusion at the top of the molecule (pyridine-R^2^) and two lobes at the left- and the right-hand side (triazole-R^1^). The different substituents R^3^ of **C1** and **C2** are clearly visible in the appearance of the AL: while **C1** exhibits a round feature next to the Pt atom stemming from the pyridine (Figure 2b), **C2** features an additional “tail” stemming from the C_5_H_11_ alkyl chain (Figure 2d–f).

We can evaluate the driving force of self-assembly and gain information about the intermolecular interactions within the first monolayer by focussing on the different packing structures. **C1** appears densely packed in a rhombic lattice with side lengths of 11.5 ± 0.3 Å and an angle of 86 ± 4° (Figure 2b). The symmetry axis of the molecule is tilted by 12.0 ± 0.5° relative to the 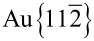 direction. However, the orientation of neighboring molecules to each other can be either parallel or antiparallel. We did not find any nearest or next-nearest neighbor correlation, i.e., the orientation in the lattice seems to be purely random. While we only found one packing for **C1**, we observed three different local patterns for **C2** within a single preparation (Figure 2c). The rectangular unit cell (Figure 2d) has a lower nominal coverage and exhibits pores with an irregular distribution. At maximum coverage the unit cell becomes oblique (Figure 2e,f). Each unit cell contains two **C2** molecules and in one case two additional elongated features (see below). The adsorption angle relative to the 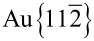 direction differs for each structure (9 ± 3° (Figure 2d), 4 ± 3° (Figure 2e) and 15 ± 3° (Figure 2f)). This is another indication for a weak overall molecule–substrate coupling. Furthermore, the lateral intermolecular interaction also seems to be relatively weak. TLs of neighboring molecules as well as the pyridine AL of complex **C1** seem to be packed in a steric fashion. However, we attribute the different patterns of **C2** to additional van der Waals forces between the amyl chains [38–39].

We note that an additional molecular structure is evident in the third pattern of **C2** (Figure 2f). Next to two rows of **C2** molecules with alternating orientation a row of paired elongated protrusions appears. This feature is quite similar in size, shape and intensity to the AL of **C2**, i.e., 4-pentylpyridine. On the other hand, isolated TLs could not be observed in any STM images. As STS spectra of these unknown elongated molecular structures remain featureless, we cannot clarify their composition or origin at this point. Nevertheless, we only observed these units at low coverages. Therefore, we suggest that a small ratio of molecules dissociates by breaking the bond between the Pt atom and the AL. This may occur when a molecule diffuses to an elbow site of the Au(111) herringbone reconstruction or a monatomic step edge, where the Au atoms have a lower coordination and hence interact stronger with adsorbates. At higher coverages, diffusion (and thus dissociation) is hindered. In fact, we cannot observe the unknown elongated molecular structures at high nominal coverages close to a complete monolayer. Occasionally, the supposed 4-pentylpyridine pairs are separated by a round protrusions. We speculate that these are Au atoms bound to the two adsorbates [40], as depicted in the model structure in Figure 2f. We note that these extra molecular structures did not have any measurable impact on the STS spectra of the **C2** complex and therefore will not be discussed any further.

### Spectroscopic analysis – molecule-substrate interactions

**Figure 3:**
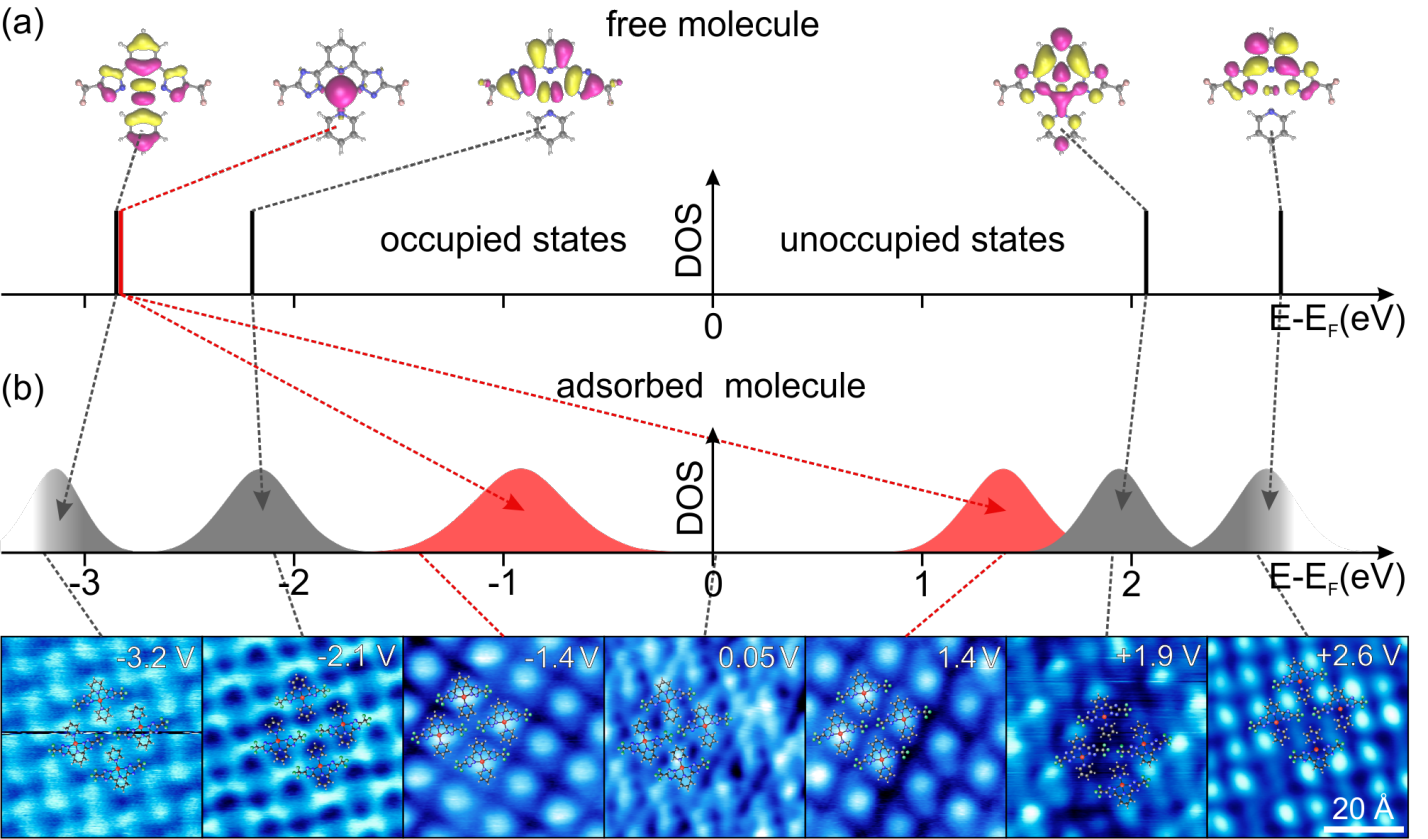
(a) Energy and LDOS of calculated orbitals for **C1** in the gas phase. Here, a work function of 5.1 eV was assumed. This value results from minimizing the energy differences between calculated and measured energies of the HOMO and the LUMO, respectively. (b) Series of *dI*/*dV* maps (bottom) and corresponding schematic representation of energetic distribution (top). Molecular states related to the Pt d_z²_ orbital are colored in red, while ligand centered orbitals are grey-shaded. The arrows between (a) and (b) indicate the orbital shifts caused by the hybridization with the substrate states.

Figure 3 shows an overview of the results from DFT calculations of **C1** in the gas phase (Figure 3a) and *dI*/*dV* maps of the first monolayer of **C1** on Au(111). The theoretical results contain the shapes and energies of five molecular orbitals with respect to *E*_F_ . While only one MO (HOMO–1) is exclusively localized at the Pt atom, all other given MOs exhibit a significant contribution at the ligands.

We first focus our discussion on the ligands (i.e., excluding the Pt site). The depicted seven *dI*/*dV* maps in Figure 3b reveal the local density of states (LDOS) of several MOs between −3.2 V and +3.2 V. Below −3.0 V the *dI*/*dV* intensity is most dominant at both pyridine rings while the triazole rings are low in intensity. Between −2.8 V and −1.8 V, the intensity distribution is reversed, i.e., triazoles now appear bright while all pyridines are dim. At positive bias voltages the triazole groups and the AL have no or weak intensities in all *dI*/*dV* maps, and features are exclusively observed at the pyridine group of the TL. Between 1.6 V and 2.3 V the signal is found centered above this pyridine ring, but above 2.3 V the *dI*/*dV* intensity is found to its left and right.

We are able to link the calculated orbitals to the measured spectroscopic maps by comparing their spatial distributions and symmetries. The calculated HOMO–2 exhibits an elongated LDOS distribution along the molecular symmetry axis with main intensities at both pyridine groups of the TL and the AL. This is in excellent agreement with the observed *dI*/*dV* maps below −3.0 V. Moreover, the HOMO shows an LDOS distribution at the two triazole groups, which is in good agreement with the experimental maps around −2.1 V. At positive energies, the calculated LUMO has an antinode along the symmetry axis of the molecule with major LDOS contribution at the TL pyridine. Again, this distribution agrees well with the spectroscopic maps seen around 2.0 V. Finally, the LUMO+1 is also localized mainly at the TL pyridine but now the orbital is antisymmetric with respect to the molecular symmetry axis. This is in very good agreement with the *dI*/*dV* distribution measured around 2.6 V. As a guide to the eye for the comparison of theory and experiment, we have schematically depicted the energetic visibility range of the described *dI*/*dV* features as Lorentzian peaks in Figure 3b that can be considered as (qualitative and schematic) experimental LDOS vs energy plots. The arrows between Figure 3a and Figure 3b display the MO assignments. As the DFT calculations only consider free molecules in the gas phase, differences originate from molecule–molecule or molecule–substrate interactions of the molecules adsorbed as a monolayer on the Au(111) surface.

We can rule out any significant lateral molecule–molecule interactions, because a comparison of **C1** and **C2** reveals virtually identical spectroscopic results despite different adsorption geometries and packing densities [30]. This also means that the molecular orientation with respect to the substrate does not seem to play a role, i.e., the overall molecule–substrate interaction cannot be large. This is indeed reflected in the above comparison. For the ligand orbitals, the major consequence of adsorbing the complex onto the Au substrate is a broadening of the levels due to weak hybridization with substrate states (i.e., physisorption) and only relatively small shifts in the energetic positions but no effect on the orbital order or occupancy.

The situation is dramatically different when focussing on the orbital features at the Pt atom. The DFT calculations show that the HOMO–1 is spatially confined to the Pt position of the complex. Further inspection reveals that this is the Pt d_z²_ state, whose lobes extend much further out of the molecular plane compared to the other frontier orbitals. In the experiment, however, we could not find any additional new feature in our spectroscopic maps between the HOMO and the HOMO–2. Instead, a spectroscopic map exhibiting intensity exclusively at the Pt site (i.e., matching the HOMO–1) was found in the range between −1.4 V and −0.4 V, i.e., higher in energy as the HOMO map! The situation is – at first glance – more confusing at positive sample bias, where we also find an identical spectroscopic map in the range from 1.0 V to 1.8 V. In fact, these two features are the first arising MOs below and above *E*_F_, respectively. In the intermediate region around *E*_F_ the *dI*/*dV* maps reflect the topographic information without a dominating contribution of a chemical group. This behavior is typical when measuring *dI*/*dV* maps within the HOMO–LUMO gap where no resonant tunneling into MO occurs [41]. Therefore, the HOMO–1 is observed twice, below and above *E*_F_. This situation is again schematically depicted in Figure 3b, where two red-colored broadened peaks represent the observed MOs at the Pt site.

**Figure 4:**
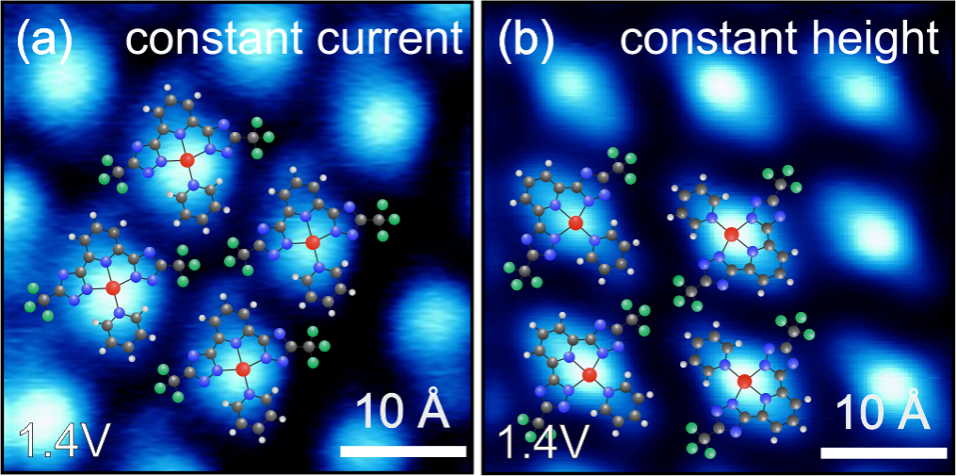
*dI*/*dV* maps of **C1** at 1.4 V recorded in constant current (a) and constant height (b) mode, respectively. 83 pA were choosen as the current setpoint.

The fact that the Pt-based state is visible in spectroscopic maps but not in local point spectroscopy should be discussed in more detail. Sometimes features observed in *dI*/*dV* maps taken in constant current mode can strongly depend on the choice of set-point current and bias, which may lead to artifacts that are not related to any electronic state [42–43]. To exclude this possibility, we additionally recorded constant-height *dI*/*dV* maps. Figure 4 shows a comparison of *dI*/*dV* maps of **C1** at 1.4 V acquired in constant-current (Figure 4a) and constant-height mode (Figure 4b). Both images show exactly the same feature with a bright protrusion at the platinum position while the rest of the molecule is low in intensity. Furthermore, we have studied four different Pt-based complexes with tremendous variations of the apparent molecular shape, and in all these complexes we observed this Pt-based spectroscopic feature. We note that it is not entirely uncommon that a spectroscopic feature might be hard to see or even entirely obscured in point spectroscopy but can be observed in *dI*/*dV* maps. This has, for instance, been found for surface states on W(110) [44] and Ni(111) [45]. Also tetracyanoethylene molecules on Cu(100) do not show any resonance in STS point spectroscopy [13], although DFT predicts at least two molecular states within the experimentally accesable energy range [46] and spectroscopic maps at *E*_F_ show the LUMO. Therefore, we conclude that the Pt-centered feature observed here is not an experimental artifact but an intrinsic and robust feature representing an orbital state.

Our observations summarized in Figure 3 can be understood when considering that the Pt d_z²_ orbital exhibits a much larger overlap with the electronic wavefunctions of the Au(111) substrate compared to the ligand orbitals (sp-like states extending less far out of the molecular plane). If the overlap is large enough, the states can hybridize, leading to a partial charge transfer from the HOMO–1 into the substrate. Essentially, two different scenarios can explain our data. In the first scenario, the hybridization may lead to a singly occupied molecular orbital (SOMO). It has been shown that a SOMO is observed twice in STS [47]: at negative bias it is probed by a tunneling process of the SOMO-electron to the tip; at positive bias a second electron is injected into the SOMO. Due to the localized nature of this MO, the Coulomb repulsion between the two electrons has to be overcome. The separation of the two peaks in Figure 3b would then correspond to the Coulomb energy. Typical Coulomb energies for d_z²_ states of organometallic complexes are around 2 eV [48–49], in agreement with the 2.4(7) eV for **C1**. In the second scenario, the Pt d_z²_ orbital may strongly hybridize with a Au state to form a (occupied) bonding and an (unoccupied) antibonding orbital [50].

**Figure 5:**
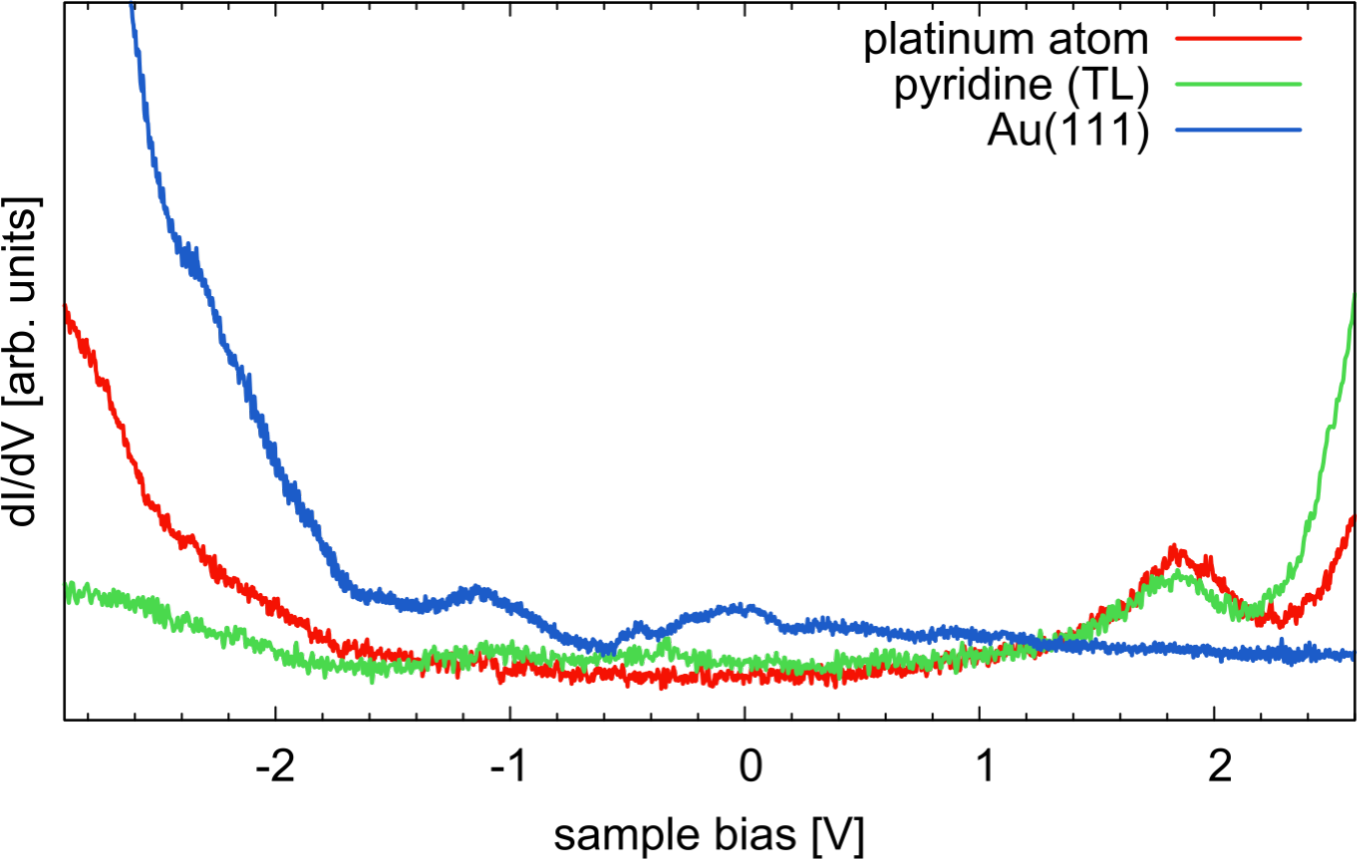
*dI*/*dV* spectra taken over the Pt atom and the pyridine group of **C1** exhibit a peak at 1.9 V that we assign to the LUMO of the free molecule (see discussion in the text). For comparison, a spectrum of the bare Au(111) surface is also shown. Results for complex **C2** are almost identical.

In order to evaluate which of the two scenarios is more likely, we can have a detailed look at the results of local STS spectroscopy (Figure 5). Spectra taken over the Pt atom only reveal a peak at about 1.9 V but no peaks around −0.9 or 1.4 V. In comparison, the spectrum on bare Au(111) is featureless at this energy, i.e., the peak is clearly a molecular state. However, it is not a state solely located at the Pt atom, because the spectrum on the pyridine group of the TL also shows a peak at the same energy and the *dI*/*dV* map at 1.9 V has almost exclusively intensity at the latter. We therefore assign the measured peak in the spectrum to the LUMO that also shows some finite contribution at the Pt site but is mainly located at the TL pyridine group (see DFT results in Figure 3a). Yet, we do not see any appearance of further peaks in the spectra between −1.5 V and 1.5 V at the Pt although the *dI*/*dV* maps unambiguously show two MO features.

Why can we observe the d_z²_ state in *dI*/*dV* maps but not in the spectra, whereas we can observe the ligand states in both measurements? For this we have to consider the possibility of strong physisorption (or even chemisorption). While weak physisorption only leads to a broadening of the MO levels (as described above), a strong molecule–substrate hybridization (i.e., strong physisorption or chemisorption) can lead to a strong broadening of a MO level as well as significant shifts in binding energy even to a degree that this state changes its occupancy due to charge transfer with the substrate [48,51–54]. For the SOMO scenario, we would expect the transfer of one electron from the Pt d_z²_ state to the Au substrate, but the emergence of a Coulomb blockade would require that the MO is still well localized and should be clearly visible as peaks in STS spectra. On the other hand, in the scenario of bonding and antibonding states the d_z²_ orbital of the Pt atom may hybridize and broaden in such a strong manner that no significant feature arises from the underground signal in the tunneling spectra. Nevertheless, in the *dI*/*dV* maps the very low intensity may still be imaged over a wide range (in our case about 1 eV). We also note that first DFT calculations including the surface do not support the formation of a SOMO. Therefore we conclude that the experimental findings are in favor of the second scenario, the formation of bonding and antibonding states.

Independent of the mechanism, we can summarize that Pt(II) complex **C1** exhibits a peculiar site-specific strong hybridization accompanied by a charge transfer that only involves the Pt atom but leaves the ligand orbitals essentially unaltered. We note that this effect has some interesting consequences in possible device applications. The shift of the Pt d_z²_ state reduces the charge-injection barrier dramatically: now the first accessible states to inject holes or electrons are not the HOMO and LUMO at about −2 and +2 eV, respectively, but the hybridized Pt MO at −0.9 and +1.4 eV. The exact energies should even be tunable in a controlled fashion by altering the degree of molecule–substrate coupling. This could be achieved by using different substrates [13,54] or alternatively by a systematic variation of the vertical Pt-substrate separation. The latter could be achieved sterically by using ligand side groups with different degrees of bulkiness. For instance, replacing the CF_3_ by *tert*-butyl or adamantyl groups [32] would lift the molecular plane further away from the substrate surface. We emphasize that our analysis of electronic properties is identical (and thus highly reproducible) for all complexes within the monolayer; i.e., the molecules interact in a well-defined way with the substrate. This is only possible due to its planar structure that leads to distinct orbital overlaps. Hence, we expect that well-defined interactions also occur in host–guest environments as well as within aggregated structures of Pt(II) complexes.

### Spectroscopic analysis - intramolecular tuning

**Figure 6:**
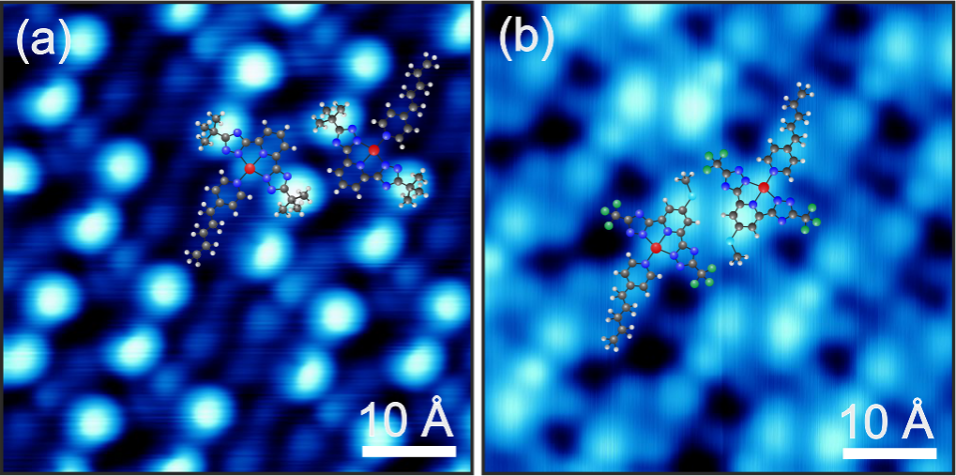
STM images of self-assembled monolayers of **C3** (a) and **C4** (b). Due to the rotational degree of freedom around the O–C bonds, the exact position of the methoxy group cannot be given here. Despite the different substituents R^1^ and R^2^ the complexes show similar packing structures indicated by the overlaid molecular models.

The electronic properties of Pt(II) complexes can, of course, also be tuned by alteration of the chemical structure. In order to understand the intramolecular interactions in more detail, we have decided to only apply a fine-tuning of the substituents. For this study we measured two modified complexes **C3** and **C4** where either R^1^ or R^2^ are substituted in comparison to **C2**. Figure 6 depicts submolecularly resolved STM images of the molecules on Au(111). Both molecules are found in self-assembled monolayer islands containing only intact molecules. The overlay of the corresponding molecular structures again allows a straight-forward identification of each molecule. **C3** (Figure 6a) exhibits two bright lobes to the left and right of a dimmer protrusion. We attribute these to the two bulky *tert*-butyl groups at R^1^ and the central Pt atom, respectively, i.e., the *tert*-butyl substituents dominate the topography. The AL and the pyridine of the TL appear as a dim elongated and round feature below and above the Pt site, respectively. In contrast, **C4** (Figure 6b) exhibits the brightest protrusion at the top of the molecule (i.e., at R^2^) where a single hydrogen atom is replaced by a methoxy group. As observed for **C1** and **C2**, the triazole groups with the CF_3_ substituent at R^1^ show only a low apparent height. The unaltered Pt atom and AL appear similar to **C2** and **C3**. The packing of both molecules in the self-assembled islands consists of interlocked double rows where R^2^ is pointing towards R^3^ of a neighboring molecule. Apart from the molecular size, the different substituents do not show any influence on the measured structures. We suggest that only weak lateral interactions, most likely van der Waals forces (especially between neighboring amyl groups), and steric effects drive the self-assembly, similar to the situation of complex **C2**.

**Figure 7:**
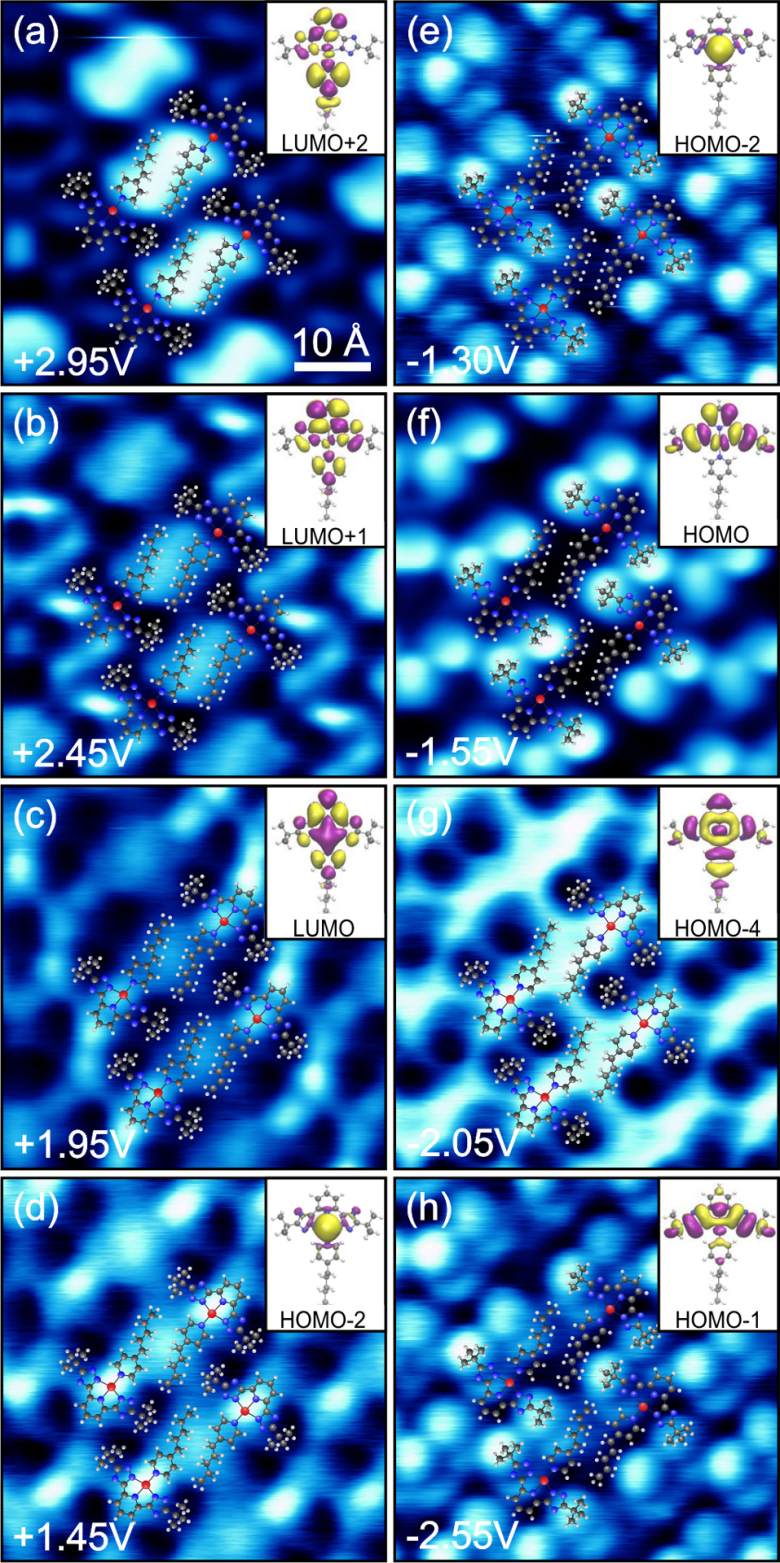
Series of *dI*/*dV* maps of complex **C3** and corresponding calculated orbitals of gas-phase molecules.

For **C3**, one may think that equally detailed STS mapping of the molecular orbitals as in Figure 3 may be prohibited by the topography-dominating *tert*-butyl groups. Nevertheless, we performed energy resolved *dI*/*dV* measurements on **C3** ranging from −2.55 V to +2.95 V. Figure 7 contains the corresponding series of *dI*/*dV* maps. An overlay of molecular structures in each map (where exact positions are again extracted from the simultaneously acquired topography images) permits the correlation of features to specific molecular parts. For comparison, the respective DFT MOs (calculated for gas-phase molecules) are reproduced in the insets. At +2.95 V (Figure 7a) a high signal is found at the AL, especially between the two neighboring amyl groups. Additionally, two lobes with low intensity show up on both sides of the TL pyridine. At +2.45 V (Figure 7b) the AL becomes dimmer while the sides of the TL pyridine are brighter. We suggest that the lack of *dI*/*dV* signal on top of the pyridine is due to a nodal line along the symmetry axis of the underlying MO. This changes at +1.95 V (Figure 7c), where a bright intensity is found above the center of the TL pyridine but not on its sides anymore. There is also a smaller signal located at the Pt atom and between the amyl groups. The situation is almost reversed at voltages of +1.45 V (Figure 7d): now the Pt position is most dominant and the top of the pyridine lost intensity. We could not find any evidence for unoccupied molecular states at the triazole groups in the given voltage range.

At negative sample bias of −1.30 V (Figure 7e) we observed waisted features at both *tert*-butyl groups and the Pt position whereas the pyridines and the amyl group are very low in intensity. The *dI*/*dV* signal at −1.55 V (Figure 7f) is concentrated at the left- and right-hand side of the TL while we do not detect any signal at the other positions. Compared to this, the *dI*/*dV* map at −2.05 V (Figure 7g) is completely inverted. Finally, at −2.55 V (Figure 7e) an asymmetric distribution of the *dI*/*dV* signal is found. The brightest features stem from the *tert*-butyl groups, but there are additional sickle-like features with lower intensity to the bottom right. The simultaneously recorded topography (not shown here) looks similar to that of Figure 6a. Therefore it is unclear whether this asymmetry is an artifact caused by the STM tip.

Essentially, despite the bulky *tert*-butyl groups, the *dI*/*dV* maps again reveal various spatial LDOS distributions that can be assigned to the simulated MOs analogous to the procedure for **C1** (Figure 5). The LUMO+2 is mainly localized at the AL and shows minor LDOS at the TL pyridine. The LUMO+1 is antisymmetric with respect to the mirror plane of the TL and has the highest LDOS at the TL pyridine. We assign these two orbitals to the features measured in the *dI*/*dV* maps at +2.95 V and +2.45 V, respectively. In the gas phase, the calculated energy levels of the LUMO+2 and LUMO+1 are quasi degenerate. Considering a much larger level broadening, we expect both MOs to be detected simultaneously but with varying relative intensity for different voltages. The symmetric LUMO exhibits a high LDOS at the pyridine extending further to the Pt atom. This is in good agreement with the map in Figure 7c. Overall, the three lowest lying unoccupied molecular orbitals reflect the results of the *dI*/*dV* measurement both in order and occupancy.

Nevertheless, the features in Figure 7d are best described by the Pt d_z²_ orbital, which is the HOMO–2 in the gas-phase calculations. This indicates a scenario analogous to **C1** where a charge transfer from the molecule to the substrate occurs. However, this does not explain why we still observe a significant *dI*/*dV* contribution at (or between) the amyl groups. This could only be understood by assuming that the LUMO+2 exhibits a relatively strong level broadening. Alternatively, an artifact of scanning the sample in constant-current mode may be possible: the signal can be increased when the tip is approached toward the substrate at these positions between two neighboring amyl groups.

At negative bias the intensities at the Pt atom and both sides of the TL (Figure 7e) can be described by a superposition of the Pt d_z²_ orbital (HOMO–2) and the antisymmetric HOMO. We suggest that the occupied MO closest to *E*_F_ is again the shifted HOMO–2 (cf. **C1**) showing a significant energetic overlap with the lower lying HOMO. In comparison to **C1** this orbital is calculated to be higher in energy which explains the simultaneous observation of both orbitals. The HOMO is individually reproduced in Figure 7f, only 0.25 eV below Figure 7e. The inverted intensity along the symmetry axis of the TL in Figure 7g is best reproduced by the calculated HOMO–4, while Figure 7h resembles the symmetry of the HOMO–1 or the HOMO–3 (not shown here) with LDOS distributed along the triazole-Pt-triazole axis. This observation provides indications for an orbital shift of the HOMO–4 towards *E*_F_.

Our analysis shows that the characterization and visualization of molecular orbitals by STS is not limited to entirely flat molecules, but can also be applied when bulky chemical groups are used. Moreover, seven different MOs were measured, which even exceeds the previous result of the flat complex (cf. Figure 3). The unoccupied MOs of **C1** and **C3** show a similar behavior and seem to be almost unaffected by the adsorption. The Pt d_z²_ orbital appears likewise below and above *E*_F_, which is why we also assume a charge transfer toward the substrate for **C3**. However, we observed an alteration of the orbital order for the occupied states of **C3**. At this point, the origin for this different behavior from **C1** is unclear, but we assume that only the MOs exhibiting a huge contribution at the Pt site (HOMO–2 and HOMO–4) are likely to be significantly influenced by the Au(111) surface, while ligand-centered states remain essentially unchanged.

**Figure 8:**
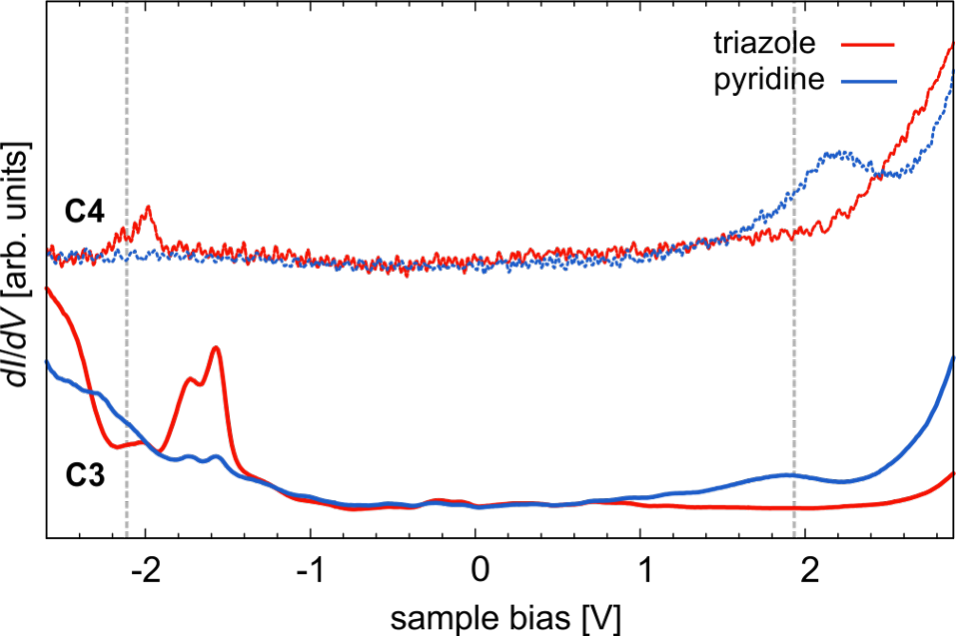
Tunneling spectra of **C3** and **C4** each acquired at the pyridine and triazole groups of the TL. For comparison, the vertical lines indicate the HOMO and LUMO levels of **C1** and **C2** [30].

Despite the substrate-induced alterations, the HOMO and LUMO orbitals of **C3** can be clearly identified. This becomes even clearer when looking at local tunneling spectra. As shown in Figure 8, a spectrum taken above the triazole group (red line) reveals a peak at about −1.5 V. This peak is almost invisible at the TL pyridine site (blue line), which confirms the strong localization of the HOMO at the triazole groups as observed in the corresponding spectroscopic map (see Figure 7f). On the other hand, the pyridine spectrum exhibits a broad peak at about 1.9 V that is not present on the triazole group. This is a clear manifestation of the LUMO orbital (Figure 7c). We can compare the HOMO and LUMO energies with those of complex **C1** and **C2** that were also quantified via local *dI*/*dV* spectroscopy [30]. We find that the LUMO level of **C3** is virtually identical to that of **C1** and **C2**, whereas the HOMO level is significantly shifted toward *E*_F_ by about 0.6 eV. In order to understand why only the HOMO level is altered, we have to discuss the effect of the different substituents. Compared to a CH_3_ or, in our case, a *tert*-butyl group, the CF_3_ group is known to have an electron-withdrawing impact on an aromatic group (here: the triazole) which can stabilize associated MOs [55–56]. The calculated frontier orbitals show that several MOs have finite LDOS at the triazole groups. However, a closer inspection reveals that the carbon atom to which the R^1^ substituents are attached only exhibits an antinode with large LDOS contribution in case of the HOMO but not the LUMO. Indeed, our spectroscopic maps of MOs show that the HOMO is localized at the triazole groups, while the LUMO shows no contribution there. Therefore it is reasonable to assume that the CF_3_ group of **C2** will only stabilize the HOMO, i.e., it shifts further away from the Fermi energy compared to **C3**. This is indeed what we see in our STS data.

We have also looked at another complex **C4** where the substituent R^2^ was changed from a hydrogen atom to a methoxy (OCH_3_) group. The latter is known to donate electrons into the π-electronic system of the attached aromatic (here: a pyridine) group, leading to a destabilization of associated unoccupied MOs. Indeed, looking at the tunneling spectra of **C4** (Figure 8), we find that the LUMO is shifted further away from *E*_F_ by about 0.4 eV, while now the HOMO is unaffected. Similar to the above discussion, this can be explained by the fact that the LUMO has a significant contribution at the TL pyridine group but none at the triazole (and vice versa). Interestingly, the DFT calculations show that both HOMO and LUMO have strong contributions at the pyridine group. However, the HOMO shows a node, the LUMO an antinode along the molecular symmetry plane, and the corresponding carbon atom of R^2^ is located right there. This subtle difference seems to decide whether the R^2^ moiety has an impact on the MO. Our finding is rather exciting as it may open the possibility to independently tune the HOMO and the LUMO levels by substitution of R^1^ and R^2^, respectively. This may have powerful consequences for OLED materials design because it should be feasible to set and tune the charge-injection barriers and the HOMO–LUMO gap, and hence the emission color, independently. We will perform further investigations on this matter to test the validity of this concept.

## Conclusion

We showed that various phosphorescent Pt(II) complexes can be deposited reliably and without dissociation onto a Au(111) surface by thermal sublimation inside an ultrahigh vacuum environment. These planar molecules are well-suited for a thorough analysis by STM and STS. We can simultaneously identify and visualize the molecular structure as well as various occupied and unoccupied molecular frontier orbitals with high submolecular spatial and meV energy resolution. We found that molecule–substrate coupling as well as specific substitution of functional groups can alter the occupation and alignment of molecular orbital levels. We emphasize the complementary benefit of combined STM and STS compared to CV or (inverse) photoemission studies whose results are often flawed by difficulties and ambiguities in the analysis due to a major lack of knowledge regarding structural integrity, homogeneity and cleanliness of samples. In a truly interlocked interdisciplinary effort, we have identified the fundamental mechanisms of external and intramolecular interactions that determine the electronic structure of the complexes, especially the HOMO and LUMO levels as well as the HOMO–LUMO gap. The results open a path toward the tailored design of triplet emitters for improving the performance of future OLED devices.
